# PPy@Fe_3_O_4_ Nanoparticles Inhibit Tumor Growth and Metastasis Through Chemodynamic and Photothermal Therapy in Non-small Cell Lung Cancer

**DOI:** 10.3389/fchem.2021.789934

**Published:** 2021-11-08

**Authors:** Danruo Fang, Hansong Jin, Xiulin Huang, Yongxin Shi, Zeyu Liu, Suqin Ben

**Affiliations:** ^1^ Department of Respiratory and Critical Care Medicine, Shanghai General Hospital, Shanghai Jiao Tong University School of Medicine, Shanghai, China; ^2^ Department of Thoracic Surgery, Shanghai General Hospital, Shanghai Jiao Tong University School of Medicine, Shanghai, China; ^3^ Department of Respiratory and Critical Care Medicine, Shanghai Jiao Tong University Affiliated Sixth People’s Hospital, Shanghai, China

**Keywords:** PPy@Fe_3_O_4_ nanoparticles, photothermal therapy, chemodynamic therapy, non-small cell lung cancer, tumor metastasis

## Abstract

Non-small cell lung cancer (NSCLC) is considered to be a principal cause of cancer death across the world, and nanomedicine has provided promising alternatives for the treatment of NSCLC in recent years. Photothermal therapy (PTT) and chemodynamic therapy (CDT) have represented novel therapeutic modalities for cancer treatment with excellent performance. The purpose of this research was to evaluate the effects of PPy@Fe_3_O_4_ nanoparticles (NPs) on inhibiting growth and metastasis of NSCLC by combination of PTT and CDT. In this study, we synthesized PPy@Fe_3_O_4_ NPs through a very facile electrostatic absorption method. And we detected reactive oxygen species production, cell apoptosis, migration and protein expression in different groups of A549 cells and established xenograft models to evaluate the effects of PPy@Fe_3_O_4_ NPs for inhibiting the growth of NSCLC. The results showed that the PPy@Fe_3_O_4_ NPs had negligible cytotoxicity and could efficiently inhibit the cell growth and metastasis of NSCLC *in vitro*. In addition, the PPy@Fe_3_O_4_ NPs decreased tumor volume and growth *in vivo* and endowed their excellent MRI capability of observing the location and size of tumor. To sum up, our study displayed that the PPy@Fe_3_O_4_ NPs had significant synergistic effects of PTT and CDT, and had good biocompatibility and safety *in vivo* and *in vitro*. The PPy@Fe_3_O_4_ NPs may be an effective drug platform for the treatment of NSCLC.

## Introduction

Lung cancer remains the most common malignant cancer, which has the highest rates of mortality. (Nasim et al., 2019; Woodman et al., 2021) Non-small cell lung cancer (NSCLC) is one of the histological subtypes, which accounts for approximately 80–85% among lung cancers. (Oser et al., 2015) Over the last decade, considerable progress has been made in the treatment of NSCLC, helping us to understand tumor biology deeply and promoting the early detection and multimodal cancer treatment. (Herbst et al., 2018) However, metastatic lung cancer is still an incurable disease and has a median survival of only 5 months. (Riihimäki et al., 2014; Rosell and Karachaliou, 2015) At present, the conservative therapeutic methods including surgery, radiotherapy and chemotherapy are efficient modes to manage advanced lung cancer, but remain unsatisfactory for improving the therapeutic efficacy. (Luo et al., 2018) As such, continuous researches on novel therapeutic compounds and combination treatments are required to amplify clinical outcomes and to improve the survival outcomes of NSCLC.

In recent years, new advances in the bioapplication of nanomaterials substantially improved the diagnosis and treatment of tumors. (Guan et al., 2021; Zhao et al., 2021) Compared with conventional inorganic nanoparticles (NPs) and small molecule-based organic nanocarriers, polymers show excellent biocompatibility, biodegradability and minimal side effects on normal tissues. (Wang, 2016) Besides, polymer chemical structures can provide various responsive components for both external stimuli [such as light, (Hribar et al., 2011) radiofrequency, (Zhang et al., 2016) ultrasound, (Paris et al., 2015) and magnetic field (Mirvakili et al., 2020)] and internal states (such as pH, (Wang et al., 2016) temperature, (Zhang et al., 2020) enzyme, (Wei et al., 2016) and redox (Zhuang et al., 2018) environment). Recently, polypyrrole (PPy) NPs have extensively investigated as powerful photothermal agents exhibiting high photothermal conversion efficiency and exceptional photostability. (Zha et al., 2013) Meanwhile, PPy NPs are easy to fabricate, with low cost and high yield. (Phan et al., 2018; Yang et al., 2018) And it is well known that iron oxide NPs are positive contrast agents for T2 weighted magnetic resonance imaging (MRI) due to their low toxicity and superior magnetic properties. (Wei et al., 2017) In this study, since Fe^3+^ ions are used as the oxidation agents to produce PPy NPs, many ferric (Fe^3+^) and ferrous ions (Fe^2+^) remain in the obtained PPy NPs. And Fe ions in the PPy NPs are selected as precursors for *in situ* formation of Fe_3_O_4_ crystals onto the surface of pre-synthesized PPy NPs. The final products (PPy@Fe_3_O_4_ NPs) possess both chemodynamic therapy (CDT) and photothermal therapy (PTT) functions, holding tremendous potential for remarkable efficiency to inhibit tumor growth. PTT, which uses photothermal agents to ablate tumors by converting absorbed light energy into intense localized heat, has attracted widespread attention as a non-invasive therapeutic technology. (Chen et al., 2019) On the other hand, CDT is an emerging therapy that generates toxic hydroxyl radicals (·OH) from endogenous hydrogen peroxide (H_2_O_2_) using Fenton/Fenton-like reaction to control tumor progression in the tumor microenvironment (TME). (Tang et al., 2019) As generally known, compared with normal cells, cancer cells have special intracellular microenvironment with higher levels of H_2_O_2_ and weak acidity, which offers prerequisites to CDT application. (Yang et al., 2017, 2019; Shen et al., 2018) Thus, PPy@Fe_3_O_4_ NPs, which have noteworthy synergistic therapeutic effects, may play different roles in the growth and metastatic of NSCLC.

Extracellular matrix (ECM) is a complex biopolymer mixture produced by different kinds of cells in the extracellular space. The ECM not only is crucial in cell adhesion and tissue organization, but also modulates cell differentiation, activation and migration. (Pickup et al., 2014; Eble and Niland, 2019) Matrix metalloproteinases (MMPs) belong to a large family of zinc-dependent endopeptidases that are involved in ECM degradation and play essential roles in cancer progression-associated pathways, including tumor growth, invasion and migration. (Gonzalez-Avila et al., 2019) Matrix metalloproteinase 2/9/13 (MMP2/9/13) are key members of the MMP family. Among these MMPs, MMP2 and MMP9 can selectively degrade type IV collagen, promoting tumor cells migrating through the basement membrane. (Wang et al., 2018) And MMP13 has the capability to degrade native collagen fibrillar types I, II, III, and VII and is related to the ECM remodeling. MMP2, MMP9 and MMP13 have been found upregulated and enhance the migration capability of lung cancer cells (Li et al., 2019; Han et al., 2020). Meanwhile, down-regulating the expression of MMP2, MMP9 aand MMP13 can reduce tumor cell growth, proliferation and metastasis (Tan et al., 2015; Poudel et al., 2016). We are wondering if PPy@Fe_3_O_4_ NPs could decrease the expression of MMP2, MMP9 and MMP13.

In this research, we examined the anti-tumor efficacy of the novel PPy@Fe_3_O_4_ NPs *in vitro* and *in vivo*. Our results displayed that the growth of tumors was considerably inhibited by the PPy@Fe_3_O_4_ NPs, and the levels of MMP2, MMP9 and MMP13 decreased. These results demonstrated that PPy@Fe_3_O_4_ NPs were excellent MRI-guided synergistic chemodynamic/photothermal cancer therapy agents and might be promising drugs to treat NSCLC because of inhibition of tumor growth and metastasis.

## Materials and Methods

### Chemicals and Reagents

Polyvinyl alcohol 1788 (PVA; Alcoholysis degree: 87.0–89.0%), iron (III) chloride anhydrous (FeCl_3_; 99.9%) and pyrrole (99%) were purchased from Aladdin chemistry Co., Ltd. (Shanghai, China). Absolute ethyl alcohol (C_2_H_5_OH; AR) and ammonium hydroxide solution (NH_3_·H_2_O; 28.0–30.0%) were purchased from Sinopharm Chemical Reagent Co., Ltd. (Shanghai, China). Deionized water (H_2_O) was prepared by a Milli-Q water purification system (Millipore, Bedford, MA, United States). All the chemical reagents were used without further purification.

### Preparation of PPy@Fe_3_O_4_ Nanoparticles

We synthesized the PPy@Fe_3_O_4_ NPs by a very facile electrostatic adsorption method. First, PVA (0.75 g) was added in the deionized water (10 ml) and heated to 95°C until the solution was entirely dissolved. Later, FeCl_3_ (0.373g, 2.30 mmol) was mixed homogeneously with the above mixture under strong magnetic stirring for 1 h. Next, pyrrole monomer (69.2μl, 0.9970 mmol) was slowly dropped, and the reaction was maintained for 4 h at 4°C under magnetic stirring. The final solution turned dark green, indicating that PPy NPs were successfully synthesized. Subsequently, the solution (2.5 ml) was taken out, mixed with deionized water (15 ml) and ethanol (2 ml) under stirring at 70°C. Afterwards, 1 ml 1.0 wt% aqueous ammonia liquid was dropped into the solution followed by stirring for 30 min. Then 1 ml of 1.0 wt% aqueous ammonia liquid was added dropwise again, and the mixture lastly maintained for 30 min at 70°C. Finally, after being washed three times by deionized water, the PPy@Fe_3_O_4_ NPs were collected by centrifugation (11000rpm) for 50 min.

### Characterization of PPy@Fe_3_O_4_ NPs

The morphology and size of PPy@Fe_3_O_4_ NPs were determined using a JEM-200 transmission electron microscopy (TEM, JEOL, Tokyo, Japan) at 200kV acceleration voltage. The characteristic functional group and crystal structures of nanomaterials were measured using Fourier-Transformed Infrared (FTIR) spectrometer and X-ray powder diffraction (XRD) respectively.

### Photothermal Effect Evaluation

Briefly, aqueous solutions of PPy@Fe_3_O_4_ NPs with different concentrations (100, 200 and 400 μg/ml) were separately irradiated by an 808 nm near-infrared (NIR) laser (1.0W/cm^2^). The temperature profiles were monitored and recorded using a thermal imaging camera (Fotric, Shanghai, China) over time. Then the PPy@Fe_3_O_4_ NPs (400 μg/ml) solutions were irradiated with the NIR laser for 10 min, and cooled down to room temperature. Last, we calculated the photothermal conversion efficiency (η) by the temperature curves according to published study. (Leng et al., 2018)

### Cytotoxicity Assay

We evaluated the cytotoxicity of the PPy@Fe_3_O_4_ NPs by cell counting kit-8 (CCK-8) assays. In detail, normal human bronchial epithelial cells (BEAS-2B) and Human lung adenocarcinoma cells (A549) were respectively seeded into 96-well plates at a density of 10^4^ cells per well and cultured in pH7.4. The following day, we replaced the medium with fresh medium (pH7.4) containing the PPy@Fe_3_O_4_ NPs of different concentrations (25, 50, 100, 200, 400 μg/ml) for another 24 h. Then we changed the medium with 100 μl serum-free medium and 10 μl CCK-8 reagent. Following incubation for 2 h, we measured the absorbance value of wells at 450 nm using a microplate reader (Thermo, United States). The calculated formulas were listed in Supplementary Material.

### ROS Detection Assay

The A549 cells were incubated in the culture plates and divided into four groups: 1) control; 2) PPy@Fe_3_O_4_ (400 μg/ml); 3) H_2_O_2_ (100 μM); 4) PPy@Fe_3_O_4_ (400 μg/ml) + H_2_O_2_ (100 μM). After 24 h of treatment, we used the probe solution 2, 7-dichlorodihydrofluorescein diacetate (DCFH-DA; Beyotime Biotechnology, Shanghai, China) to estimate the intracellular reactive oxygen species (ROS). After DCFH-DA treatment for 20 min, the cells were washed three times by phosphate buffered saline (PBS) buffer and blank medium was added. Finally, we acquired images using Confocal laser scanning Microscopy (CLSM, Leica Microsystems, Mannheim, Germany).

### Anticancer Effect *in vitro*

The anti-cancer effect of PPy@Fe_3_O_4_ NPs was evaluated by the Calcine-AM/propidium iodide (PI) test (Beyotime Biotechnology, Shanghai, China) and the Annexin V-FITC/PI apoptosis kit (Multi Sciences, Hangzhou, China). To investigate the PTT effect of the PPy@Fe_3_O_4_ NPs, A549 cells were incubated in the culture plates at pH6.5 and divided into 6 groups; group 1 with cells only; group 2 cultured with NIR only; group 3 cultured with H_2_O_2_ (100 μM) only; group 4 cultured with PPy@Fe_3_O_4_ NPs (400 μg/ml) + NIR; group 5 cultured with PPy@Fe_3_O_4_ NPs (400 μg/ml) + H_2_O_2_ (100 μM); group6 cultured with PPy@Fe_3_O_4_ NPs (400 μg/ml) + H_2_O_2_ (100 μM) + NIR. After incubation with PPy@Fe_3_O_4_ NPs (400 μg/ml) for 12 h, the cells of group 2,4 and 6 were exposed to an 808 nm NIR laser (1.0 W/cm^2^) for 10 min. Afterwards, we stained the A549 cells with Calcein-AM and PI for about 15 min, and images were acquired by CLSM.

And we detected cell apoptosis by Annexin V-FITC Apoptosis kit. The A549 cells in different groups were harvested and washed with PBS and Binding Buffer. Then the cells were resuspended in 500 μl Bind Buffer with 5 μl Annexin V-FITC and 10 μl PI solution to mix evenly for 5 min at room temperature in darkness. And we measured cell apoptosis immediately by flow cytometry (cytoflex LX, Beckman Coulter).

### Transwell Migration Assay

*In vitro* migration assay, 24-well Transwell chambers with a polycarbonate filter membrane of 8 μm pore size (Corning, United States) were used to assess the effect of the PPy@Fe_3_O_4_ NPs on A549 cell migration. Cells were divided into control group and PPy@Fe_3_O_4_ group. After starvation of A549 cells for 24 h, we resuspended cells in serum-free RPMI 1640 medium and serumfree RPMI 1640 medium containing the PPy@Fe_3_O_4_ NPs at a concentration of 400 μg/ml respectively. Next, the A549 cells were inoculated into the upper chamber and the RPMI 1640 medium was placed in the lower chamber. After culturing for 24 h, the A549 cells that migrated to the bottom of the membranes were immobilized and stained with 1% crystal violet (Beyotime Biotechnology, Shanghai, China). Finally, the migrated cells of each groups were counted under a microscope (Leica, Germany).

### Western Blotting

A549 cells (5*10^5^) were seeded into 6-well plate and divided into the control group and the PPy@Fe_3_O_4_ group (400 μg/ml). After incubation for 24 h, the cells were washed by PBS and lysed in RIPA buffer. Then we removed the cell debris by centrifugation, and the supernatants were harvested and stored at −80°C. Equivalent amounts of protein (25 µg) were separated on 10% SDS-PAGE and transferred onto 0.22 um polyvinylidene difluoride (PVDF) membranes. The membranes were blocked and subsequently incubated with primary antibodies including anti-MMP2/MMP9/MMP13 (Abclonal, China, 1:1,000 dilution) and anti-β-actin (Proteintech, China, 1:10,000 dilution). The following day, we washed the membranes three times for 10 min each time by Tris-buffered saline with Tween20 (TBST), and then incubated them in the corresponding secondary antibody (goat anti-rabbit IgG horseradish peroxidase (HRP), goat anti-mouse IgG-HRP, 1:1,000, Affinity Biosciences, China) at room temperature for 2 h. Following washing three times for 10 min with TBST buffer, the membranes were treated with enhanced chemiluminescence (ECL, EpiZyme, Shanghai, China) reagent for exposure.

### Magnetic Resonance Imaging *in vivo*

For *in vivo* MRI measurements, we intratumorally injected the A549 tumor-bearing mice with 200 μl of 3 mg/ml PPy@Fe_3_O_4_ NPs, when tumor size reached visible size (7–9 mm in diameter). Then we scanned the mice before and 3 h after injection. Thus, we successfully acquired the high-resolution T2-weighted MRI scan images of mice by a 3.0T MRI system (Ingenia 3.0T CX, Philips Healthcare). The T2-weighted MRI parameters were as follows: pulse waiting time (TR) = 2,800 ms, echo time (TE) = 60 ms, slice width (SW) = 5.0 mm.

### Tumor Treatment *in vivo*

When the tumors reached 7–9 mm in diameter, we divided the mice into four groups (n = 6 per group): 1) control; 2) PBS + NIR; 3) PPy@Fe_3_O_4_ NPs (3 mg/ml); 4) PPy@Fe_3_O_4_ NPs (3 mg/ml) + NIR. The mice of group3 and 4 were injected intravenously with 200ul PPy@Fe_3_O_4_ NPs (3 mg/ml) and the mice of group2 was injected intravenously with 200ul PBS. Then 8 h after injection, the mice of group2 and 4 were irradiated with 808 nm laser (1W/cm^2^) for 10 min. Meanwhile, we measured the temperature changes of the tumor by a FLIR A300 thermal imaging camera. Simultaneously, tumor volume and body weight were monitored every 2 days. On day 14, the mice were sacrificed. Then the major organs were isolated for photograph or histological analysis (hematoxylin and eosin (H and E) staining), and the tumor was stained subsequently by immunohistochemistry (Ki67) and immunofluorescence (TUNEL).

### Immunohistochemistry and Immunofluorescence

The tumor tissues specimens of mice were fixed in 4% paraformaldehyde for 24 h and embedded in paraffin. Then the tissue blocks were cut into sections of 3-5 um thickness. For Ki67 staining, the sections were immunostained overnight at 4°C with an anti-Ki67 antibody (Abcam). After washing in PBS, the sections were subsequently incubated with the second antibody for 1 h. Then the slides were stained with 3, 3-diaminobenzidine (DAB) and hematoxylin separately, dehydrated, and mounted. Images were captured using a microscope (Leica, Leica DMi8). For TUNEL staining, the proportion of apoptotic cells were performed by terminal deoxynucleotidyl transferase-mediated dUTP nick end labeling (TUNEL) staining kit (Servicebio, Wuhan, China). We obtained all images using a CLSM.

### Statistical Analysis

All results and measurements were shown as the mean ± SD (standard deviation). Comparison between the mean values of different groups were analyzed by one way analysis of variance (ANOVA) or Student’s t-test: (*) *p* < 0.05 was considered statistically significant; (**) *p* < 0.01 and (***) *p* < 0.001 was considered highly statistically significant; (****) *p* < 0.0001 was considered extremely statistically significant.

## Results

### Synthesis and Characterization of the PPy@Fe_3_O_4_ NPs

**FIGURE 1:**
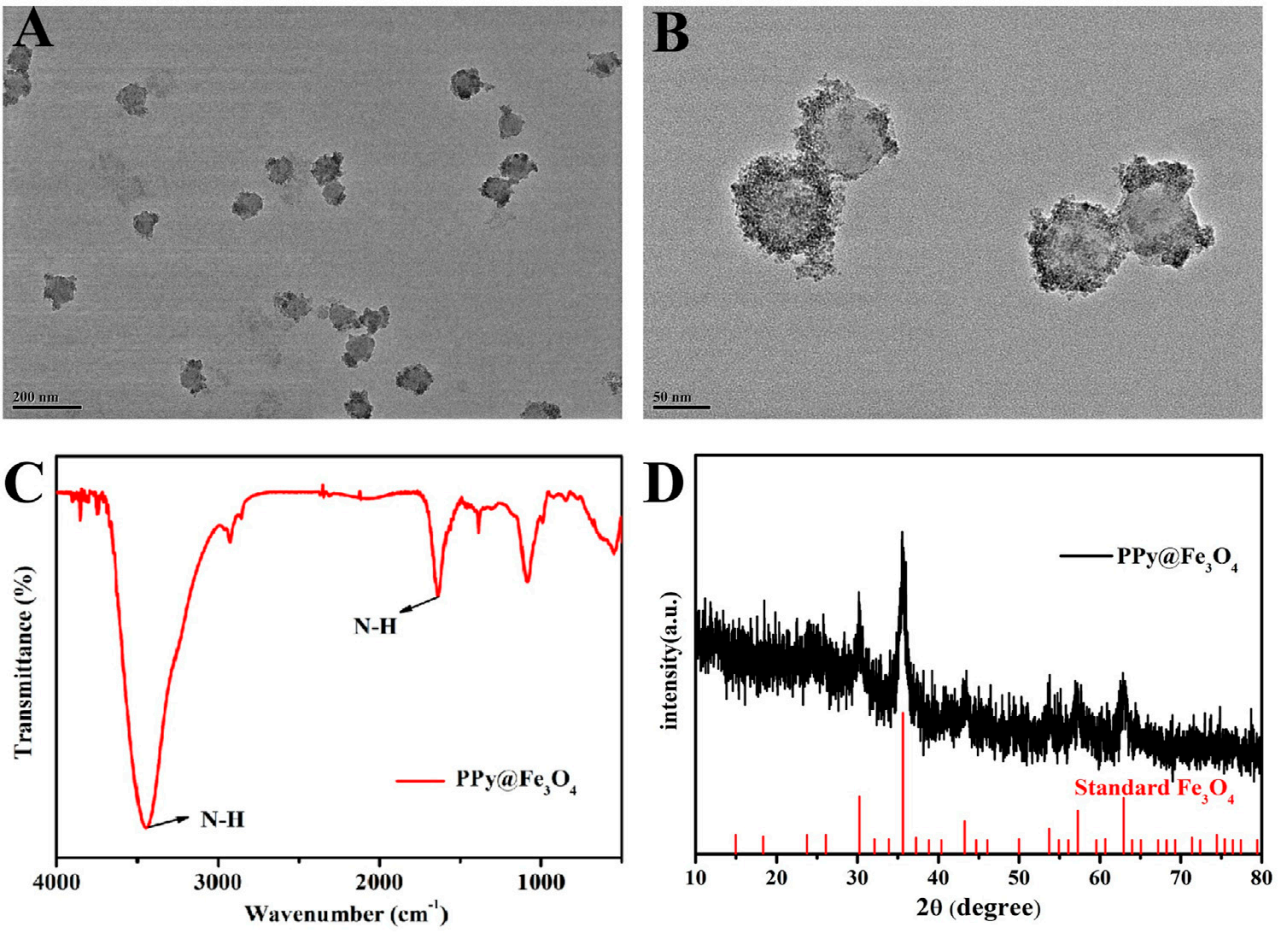
The characterization of PPy@Fe_3_O_4_ NPs. **(A)** Low- and **(B)** medium-magnification TEM image of the PPy@Fe_3_O_4_ NPs. **(C)** FTIR spectra of PPy@Fe_3_O_4_ NPs. **(D)** XRD spectra of PPy@Fe_3_O_4_ NPs.

We used TEM to determine the sizes and morphologies of PPy@Fe_3_O_4_ NPs. The PPy@Fe_3_O_4_ NPs are shown in Figure 1A, Figure 1B and Supplementary Figure S1. As we can see, the mean diameter of PPy@Fe_3_O_4_ NPs was measured to be 71.0 nm, and many tiny Fe_3_O_4_ nano-crystals were surface adsorbed on PPy NPs. The FTIR spectrum showed clearly the characteristic absorption peaks of PPy, indicating the successful formation of PPy (Figure 1C). And XRD patterns showed that the PPy@Fe_3_O_4_ NPs had characteristic peaks (Figure 1D), which could be indexed to the Fe_3_O_4_ nanocrystals (JCPDS No. 65–3,107). (Wang et al., 2020) The above results demonstrated that the PPy@Fe_3_O_4_ NPs had been successfully prepared.

### Photothermal Performance of PPy@Fe_3_O_4_ Nanaparticles

**FIGURE 2:**
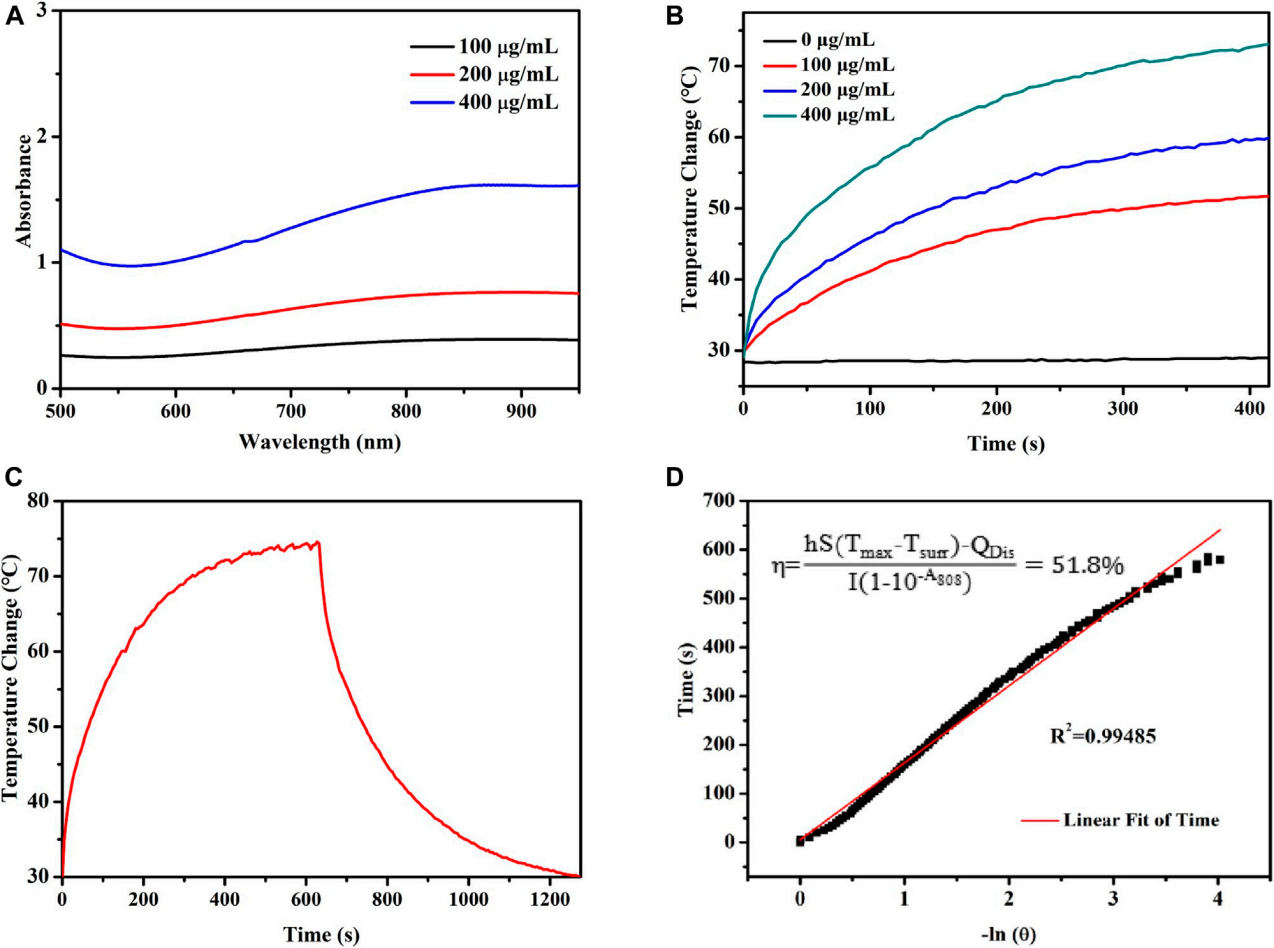
Evaluation of the photothermal properties of the PPy@Fe_3_O_4_ NPs. **(A)** UV-Vis-NIR absorption spectra of PPy@Fe_3_O_4_ NPs. **(B)** Temperature changes of PPy@Fe_3_O_4_ NCs at varying concentrations for 5 min with laser irradiation. **(C)** The curves of temperature increase with irradiation and nature cooling for PPy@Fe_3_O_4_ NCs solution (400 μg/ml). **(D)** Linear regression with a system time constant of the cooling curve shown in **(C)**.

As shown in Figure 2A, the aqueous dispersion of PPy@Fe_3_O_4_ NPs had a broad absorption throughout the visible to the NIR region, which demonstrated that PPy@Fe_3_O_4_ NPs were good potential PTT agents. We further evaluate the photothermal properties of PPy@Fe_3_O_4_ NPs with NIR light irradiation. With the prolonging of the irradiation time and increased concentration of PPy@Fe_3_O_4_ NPs, the temperature rose rapidly (Figure 2B). Finally, when the concentration of PPy@Fe_3_O_4_ NPs reached 400 μg/ml, the temperature reached about 70°C after 5 min of irradiation, which was high enough for irreversible tumor ablation (Figure 2C). Next, we tested the photothermal conversion efficiency (ŋ) of PPy@Fe_3_O_4_ NPs, which can show the capability of converting energy of light into heat. As shown in Figure 2D, the ŋ value of PPy@Fe_3_O_4_ NPs was figured out to be ∼51.8%, which is much higher than traditional PTT agents, such as Cu_2-x_Se nanocrystals, Cu_9_S_5_ nanocrystals and Au nanorods. (Hessel et al., 2011; Tian et al., 2011)

### *In vitro* Cell Cytotoxicity Assay

**FIGURE 3:**
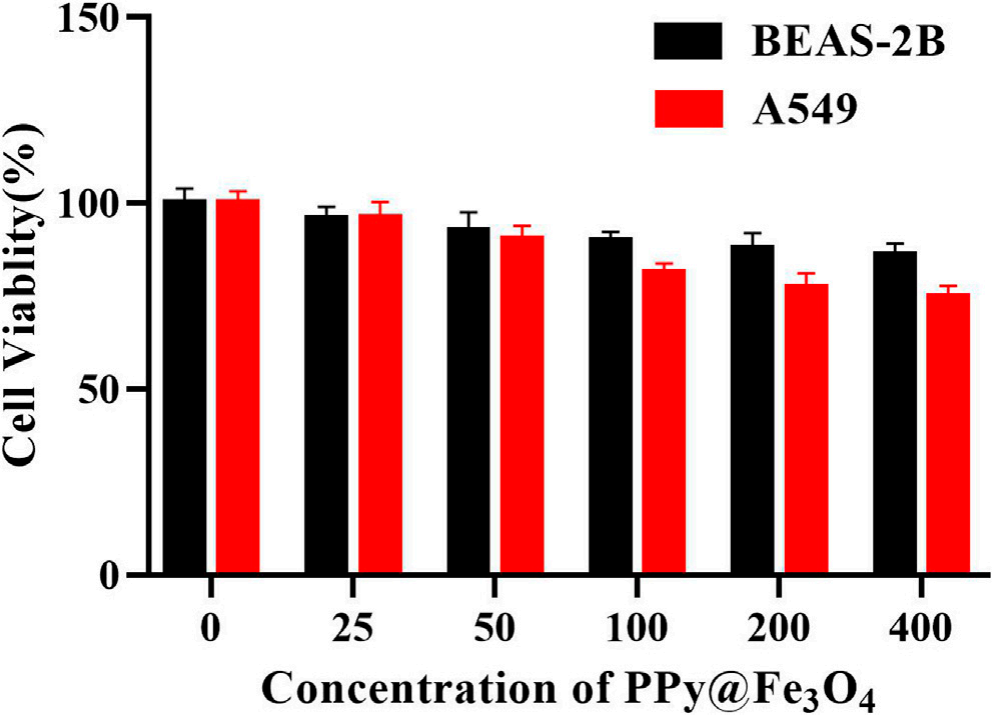
Cell viability of A549 cells and BEAS-2B cells cultured with PPy@Fe_3_O_4_ NPs for 24 h at different concentrations.

To assess the cytotoxic effects of PPy@Fe_3_O_4_ NPs, the CCK8 assay was performed on BEAS-2B and A549 cells. The cells were incubated with PPy@Fe_3_O_4_ NPs (0, 25, 50, 100, 200, 400 μg/ml) for 24 h to test the cell viability. The results indicated that PPy@Fe_3_O_4_ NPs exhibited lower cytotoxicity towards normal and cancer cells as high as 400 μg/ml (Figure 3), demonstrating that they have good biocompatibility.

### The PPy@Fe_3_O_4_ NPs Increase Intracellular ROS Generation Induced by H_2_O_2_ and Induce Cell Apoptosis

**FIGURE 4:**
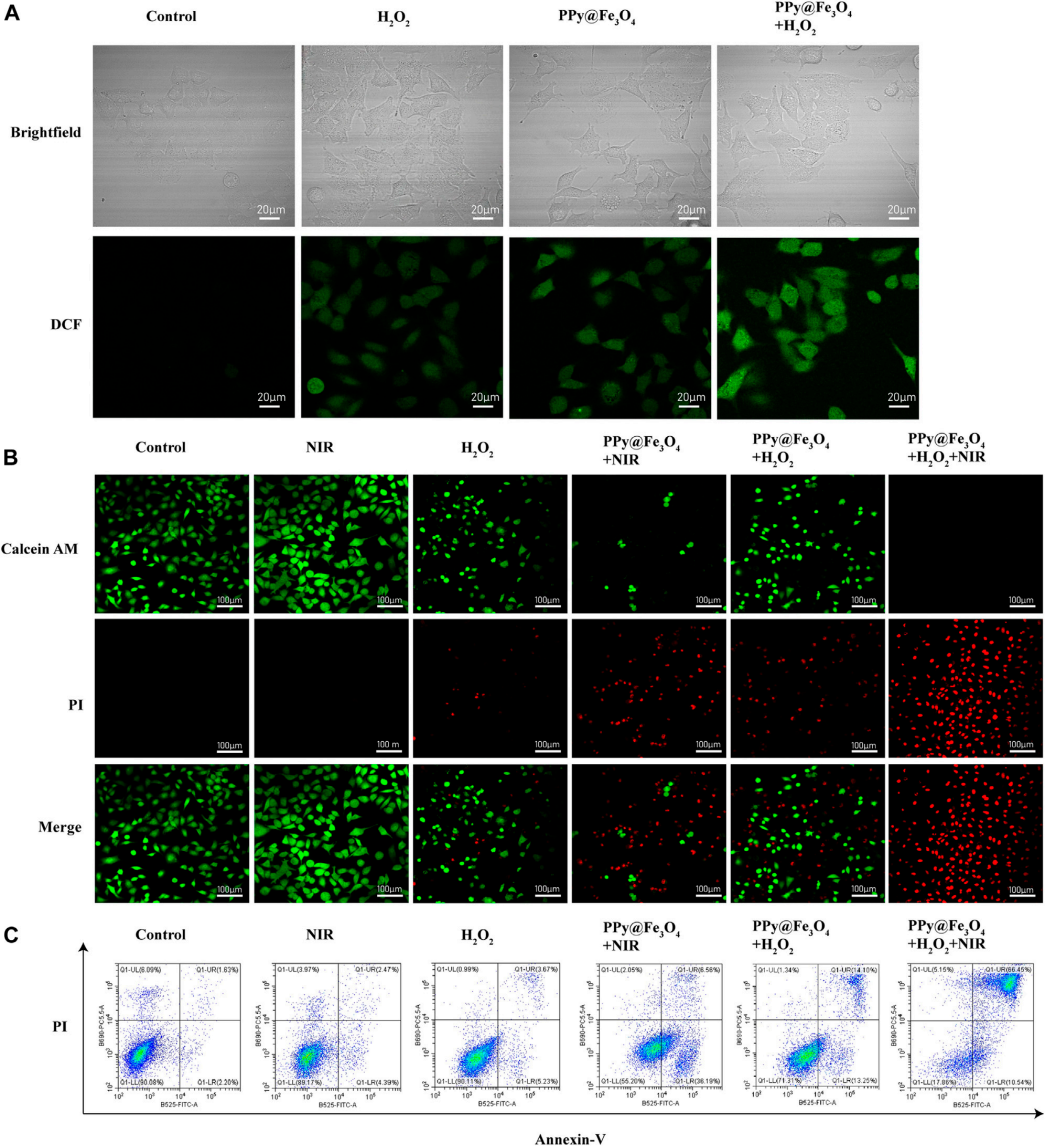
The PPy@Fe_3_O_4_ NPs increase intracellular ROS generation induced by H_2_O_2_ and induce cell apoptosis. **(A)** Brightfield images and confocal images of ROS level in A549 cells. **(B)** Confocal images of Calcein-AM and PI co-stained A549 cells in different groups. Scale bar, 100 μm. **(C)** Cell apoptosis measured by flow cytometry.

Chemodynamic therapy is an effective therapeutic treatment that causes damage of tumor cells by producing ROS. To examine the effects of PPy@Fe_3_O_4_ NPs on ROS production, we detected ROS by fluorescent probe 2′, 7′-dichlorofluorescein-diacetate (DCFH-DA), which was oxidized to 2, 7′-dichlorofluorescein (DCF) in the presence of ROS. As displayed in Figure 4A, compared with control group, the intensity of DCF fluorescence in H_2_O_2_ group and PPy@Fe_3_O_4_ NPs group increased weakly. When treated with both H_2_O_2_ and PPy@Fe_3_O_4_ NPs, the cells displayed strong and extensive green fluorescence, indicating that PPy@Fe_3_O_4_ NPs could increase Fenton-induced ROS generation in the presence of H_2_O_2_.

Given the desirable photothermal conversion performance and significant synergistic effects, we further determined the *in vitro* therapeutic efficacy of PPy@Fe_3_O_4_ NPs through Calcine-AM/PI test. Living cells were stained with green fluorescent calcein AM, while dead cells were stained with red fluorescent PI (Figure 4B). Compared with the control group, A549 cells exhibited strong green fluorescence and no red fluorescence under exposure of NIR laser radiation, which indicated that the viability of the cells was not compromised. In contrast, H_2_O_2_ slightly increased the percentage of dead cells. And A549 cells showed a remarkable cell death after treatment by PPy@Fe_3_O_4_ NPs with NIR irradiation or PPy@Fe_3_O_4_ NPs with H_2_O_2_, suggesting that the photothermal therapy and chemodynamic therapy of the PPy@Fe_3_O_4_ NPs could effectively killed the A549 cells. When A549 cells were treated by the combination of PTT and CDT, very few living cells were observed. To sum up, PPy@Fe_3_O_4_NPs have satisfactory synergistic therapeutic effects.

We measured the influence of PPy@Fe_3_O_4_ NPs on cell apoptosis by using Annexin V/PI staining and flow cytometry (Figure 4C, Supplementary Figure S2). The early apoptosis cells were defined as Annexin V (+)/PI (−), and cells in the late stage of were considered as Annexin V (+)/PI (+). The flow cytometry results showed that NIR group had no evident difference compared to the control group. Nevertheless, after treatment with H_2_O_2_, the percentage of apoptosis cells increased to 8.9%. After treatment by PPy@Fe_3_O_4_ NPs with NIR irradiation and PPy@Fe_3_O_4_ NPs with H_2_O_2_, the percentage of apoptosis cells significantly increased to 42.75% and 27.35%. And most surprisingly, the apoptosis rate in the PPy@Fe_3_O_4_ NPs + H_2_O_2_ + NIR group increased highly to 76.99%, which further demonstrated that the PPy@Fe_3_O_4_ NPs + H_2_O_2_ + NIR group had the highest killing effect due to synergistic PTT and CDT.

### The PPy@Fe_3_O_4_ Nanoparticles Inhibit the Migration of Lung Cancer Cells and Decrease MMP2/MMP9/MMP13 Expression Levels

**FIGURE 5:**
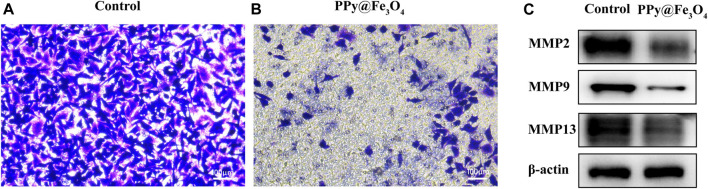
The PPy@Fe_3_O_4_ NPs inhibit the migration of human lung cancer cells. **(A,B)** The migration capacity of A549 cells measured using transwell assays after PPy@Fe_3_O_4_ NPs (400 μg/ml) treatment for 48 h. Scale bar, 100 μm. **(C)** Protein levels of MMP2/MMP9/MMP13 in different groups.

To assess cell mobility in response to the PPy@Fe_3_O_4_ NPs, we used transwell cell cultures *in vitro*, which certified the effect of PPy@Fe_3_O_4_ NPs on suppressing the migration of lung cancer cells (Figures 5A,B).

MMP2/9/13 had been found to play vital functions in the invasion and metastasis of malignant tumors. Therefore, we examined the impact of PPy@Fe_3_O_4_ NPs on these protein expressions in A549 cells using western blotting (Figure 5C). The outcomes showed that the expressions of MMP2, MMP9 and MMP13 were obviously declined in the PPy@Fe_3_O_4_ NPs group compared to the control group. In summary, these experiments suggested that PPy@Fe_3_O_4_ NPs could suppress the growth and metastasis of A549 cells.

### The PPy@Fe_3_O_4_ Nanaparticles Inhibit the Growth of Xenograft Tumors in Nude Mice

**FIGURE 6:**
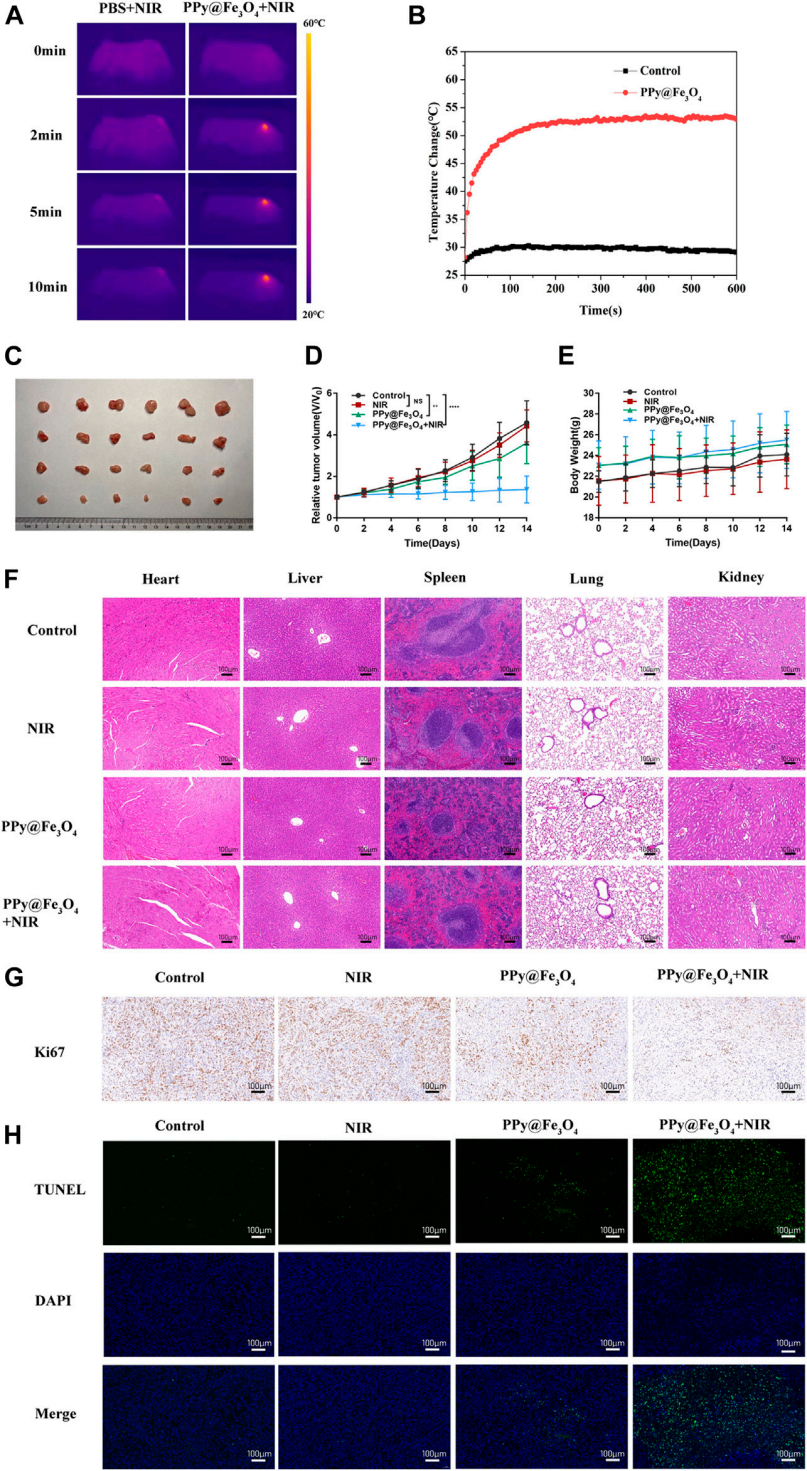
The PPy@Fe_3_O_4_ NPs inhibit the growth of tumor xenografts in nude mice. **(A)** Infrared thermography pictures of A549 tumor-bearing mice treated with PBS and PPy@Fe_3_O_4_ NPs under irradiation (808 nm, 1.0 W/cm^2^). **(B)** Profile of temperature variation of the tumor areas. **(C)** Tumor photo excised on day 14 after treatments. **(D)** Relative tumor volume and **(E)** body weight of mice in different groups. **(F)** H&E staining of main organs collected from mice in different groups. Scale bar, 100 μm. **(G)** Representative immunohistochemical stain images for Ki67 in xenograft tumors. Scale bar, 100 μm. **(H)** Representative TUNEL stain images in xenograft tumors. Blue color: DAPI staining for cell nuclei. Green color: positive apoptotic cells. Scale bar, 100 μm.

To identify the therapeutic efficacy of PPy@Fe_3_O_4_ NPs on tumor growth *in vivo*, we conducted further comparative studies. 1 × 10^6^ A549 cells were subcutaneously injected into BALB/c nude mice. When the volumes of tumors reached to approximately 100 mm^3^, we divided the mice into four groups randomly: 1) control; 2) PBS + NIR; 3) PPy@Fe_3_O_4_ NPs; 4) PPy@Fe_3_O_4_ NPs + NIR. Mice of the group 2 were injected with 200ul PBS via the tail vein, and mice of group 3, 4 were injected with PPy@Fe_3_O_4_ NPs (3 mg/ml) via the tail vein. After 8 h, the mice of group 2, 4 were irradiated by an 808 nm NIR laser (1.0W/cm^2^) for 10 min. Meanwhile, by using an infrared thermal camera, we found that the tumor temperature of mice injected with PPy@Fe_3_O_4_ NPs rapidly increased to ∼53°C during irradiation, which was sufficient for ablating tumors. However, the tumor temperature of the group 2 had negligible rise (Figures 6A,B). After 14 days, the mice were sacrificed and the tumor volumes and body weights were recorded (Figures 6C–E). The growth of tumors in group 2 was similar to the control group, demonstrating that NIR alone could not inhibit tumor growth. Whereas, tumor growth in the group 3 was partially inhibited due to CDT of PPy@Fe_3_O_4_ NPs. Noticeably, the tumor growth in group 4 was substantially inhibited because of the synergistic effects of PTT and CDT. Representative pictures of the tumors in different groups further determined the therapeutic efficacy. There was no obvious difference in body weight among the groups, suggesting that the adverse effect of PPy@Fe_3_O_4_ NPs was negligible. Additionally, the main tissues and organs of different groups were collected for H&E staining to estimate the safety of various treatments (Figure 6F). We could not find evident damage in the group 2, 3, 4, compared to the control group, illustrating that the PPy@Fe_3_O_4_ NPs were biologically safe *in vivo*.

Next, we evaluated the proliferation and apoptosis of tumor cells by immunocytochemistry and immunofluorescence (Figures 6G,H). We noticed that PPy@Fe_3_O_4_ NPs could slightly reduce the expression of the proliferation marker Ki67 and increase apoptosis, compared with the control group. Moreover, treatment of PPy@Fe_3_O_4_ NPs with NIR further significantly decreased the level of Ki67 and caused cell apoptosis more effectively. These results clearly showed that PPy@Fe_3_O_4_ NPs with NIR could suppress effectively tumor growth *in vivo*.

### The PPy@Fe_3_O_4_ Nanoparticles can Serve as Magnetic Resonance Imaging Contrast Agents to Detect Tumors

**FIGURE 7:**
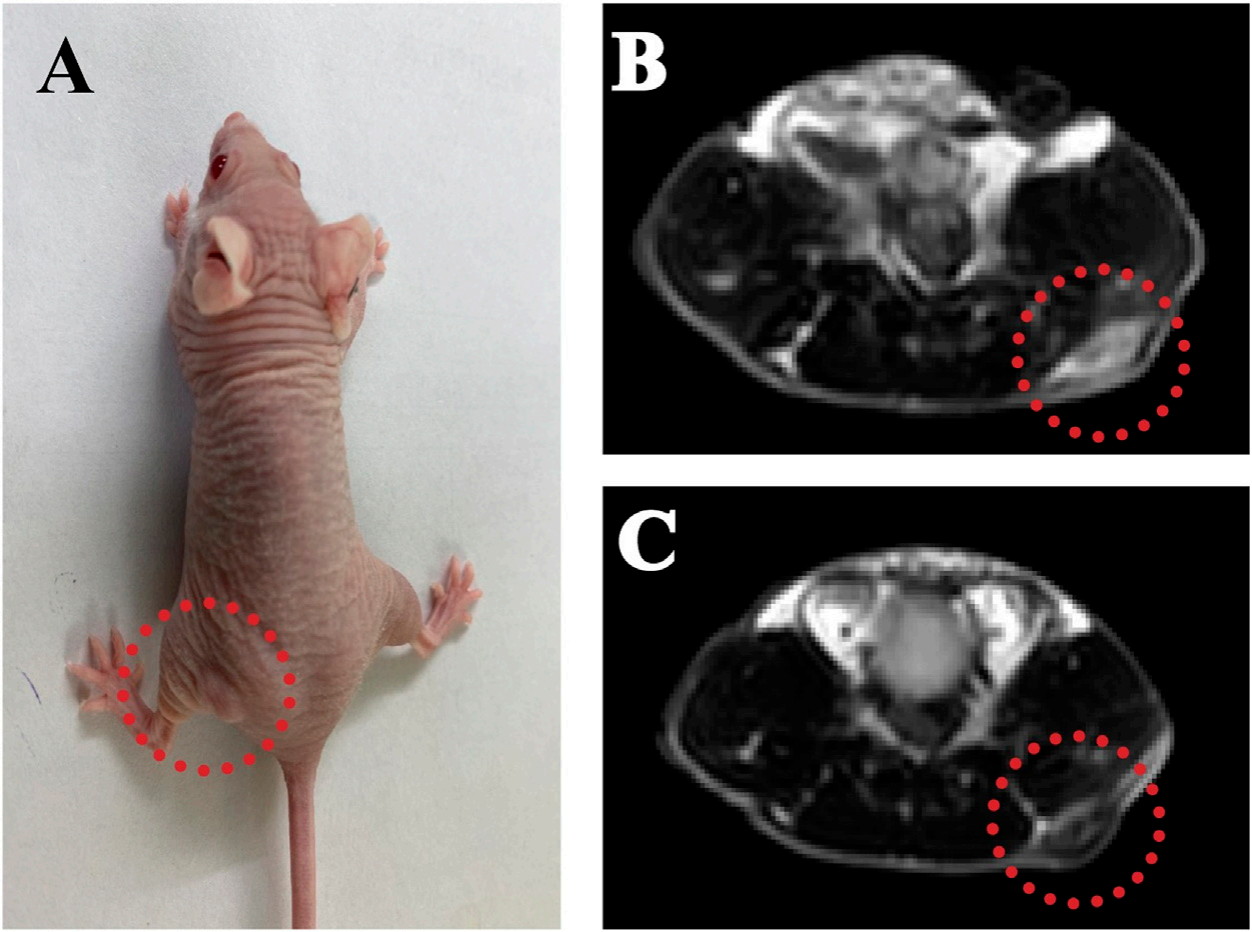
The PPy@Fe_3_O_4_ NPs can serve as MRI contrast agents to detect tumors. **(A)** Representative MRI images of A549 tumor-bearing mice; **(B)** T2-weighted MRI before and **(C)** after injection of PPy@Fe_3_O_4_ dispersions.

MRI offers excellent spatial resolution and tissue penetration, which is a noninvasive method for the identification and early cancer diagnosis. We used PPy@Fe_3_O_4_ NPs as contrast agents to evaluate the contrast-enhancing effect *in vivo* (Figure 7A). We treated the tumor-bearing mice with the PPy@Fe_3_O_4_ NPs by intratumor injection, and utilized T2 weighted MRI to observe the tumor before PPy@Fe_3_O_4_ NPs injection and 2 h post-injection (Figures 7B,C). Compared with pre-injection, we revealed that the signal intensity of tumor sites changed obviously 2 h after injection, which indicated that the PPy@Fe_3_O_4_ NPs might be ideal contrast agents for MRI in future applications.

## Discussion

For a long time, NSCLC has been a type of disease characterized by late diagnosis and resistance to therapy. (Hirsch et al., 2017) The greatest challenge in the modern nanomedicine of lung cancer is to develop promising tools for diagnosing and treating the advanced NSCLC owing to the rapid metastasis, which is the major impediment of successful treatments in the clinical procedures. Encouragingly, in this study, the PPy@Fe_3_O_4_ NPs showed satisfactory safety and efficacy as eminent photothermal agents for NSLC therapy. Additionally, we revealed that PPy@Fe_3_O_4_ NPs could suppress the proliferation, migration and metastasis of cells of NSCLC.

Polypyrrole nanoparticle, a flexible and conductive polymer, is a recognized valuable multipurpose material due to its conductive properties, outstanding stability and high absorbance in the NIR region. Therefore, it can be used in various biomedical fields, especially in the therapy of cancer. (Chiang and Chuang, 2019) Nevertheless, because of some shortcomings of PPy, such as insolubility to water, the photothermal applications of PPy-based nanoparticles are still in the development. (Chen and Cai, 2015; He et al., 2018) For overcoming these shortcomings, we formed Fe_3_O_4_ crystals onto the surface of PPy NPs in this study. Fe_3_O_4_ greatly improved the solubility and could be used as T2 contrast agents in MRI to provide diagnostic information. (Wang et al., 2012; Wei et al., 2017) And we disclosed the synergistic therapeutic effects of PPy@Fe_3_O_4_ NPs, including PTT and CDT. PPy@Fe_3_O_4_ NPs converted NIR light energy into heat energy and initiated the Fenton reaction under the mildly acidic conditions of the TME. Therefore, the apoptosis of cells would be induced and the growth and metastasis of cells would be inhibited.

In PTT, different kinds of photothermal agents contribute to the localized heating of cells and tissues. When irradiated by the light of appropriate wavelength, these agents absorb the photon energy and the singlet ground state could be converted to a singlet excited state. The excited electrons can go back to the ground state through vibrational relaxation, mediated by the interaction between the excited molecules and the surrounding molecules. Thus, the heating of the surrounding microenvironment appears due to increased kinetic energy. (Li X. et al., 2020) When the temperature reaches a certain value, cell necrosis will happen. (Knavel and Brace, 2013) Considering the high NIR photothermal conversion efficiency of PPy@Fe_3_O_4_ NPs, we performed a series of experiments to assess whether PPy@Fe_3_O_4_ NPs could be potential photothermal agents to inhibit the proliferation of lung cancer cells. We first studied the cytotoxicity of PPy NPs by CCK8 assay *in vitro* and the outcomes showed that the PPy@Fe_3_O_4_ NPs had negligible cytotoxicity to BEAS-2B and A549 cells as high as 400 μg/ml. Additionally, our study showed that administration of the PPy@Fe_3_O_4_ NPs did not induce damage to major organs, which reflected the safety of the PPy@Fe_3_O_4_ NPs *in vivo*. The Safety and efficiency of PPy@Fe_3_O_4_ NPs demonstrated that the PPy@Fe_3_O_4_ NPs might be perfect candidates for further clinical application of lung cancer. Then Annexin V-FITC/PI assay and Calcine-AM/PI test were performed to observe apoptosis induced by the PTT of PPy@Fe_3_O_4_ NPs. The results indicated that PPy@Fe_3_O_4_ NPs had strong apoptosis-inducing effects on A549 cells under the condition of NIR irradiation. For *in vivo* experiments, the data showed that PPy@Fe_3_O_4_ NPs under the NIR irradiation condition obviously caused severe tumor damage while the adjacent normal tissue would not be affected. Meanwhile, the results of the Ki67 and TUNEL assay also demonstrated that PTT of PPy@Fe_3_O_4_ NPs could inhibit growth and induce apoptosis of cells.

Chemodynamic therapy, which is regarded as *in-situ* treatment by Fenton and Fenton-like reactions, has received an increasing amount of attention. (Min et al., 2020) The highly oxidative •OH or O_2_ generated from Fenton and Fenton-like reactions, can be stimulated by the endogenous H_2_O_2_ of cancer cells and catalyzed by transition metal ions or their complexes. (Li Y. et al., 2020) Briefly, PPy@Fe_3_O_4_ NPs dissolve ferrous ions under the mildly acidic conditions of the TME and activate the Fenton reaction. The Haber–Weiss reaction is as follows:

$${\text{Fe}}^{2+}+{\text{ H}}_{2}{\text{O}}_{2}\to {\text{Fe}}^{3+}+•\text{OH}+{\text{OH}}^{-}$$ (1)

$${\text{Fe}}^{3+}+{\text{ H}}_{2}{\text{O}}_{2}\to {\text{Fe}}^{2+}+•\text{OOH}+{\text{H}}^{+}$$ (2)

$${\text{Fe}}^{3+}+\;•\text{OOH}\to {\text{Fe}}^{2+}{+\text{O}}_{2}+{\text{H}}^{+}$$ (3)

On the one hand, excessive production of •OH can oxidize vital cellular constituents, such as DNA, proteins and lipids, which can induce cell apoptosis or necrosis. (Goldstein et al., 1993) On the other hand, O_2_ production mediated by Fenton reaction can alleviate tumor hypoxia, which may improve cancer therapeutic combination strategies. (Ma et al., 2016) As we all know, hypoxia is a crucial driver to drug resistance in tumor therapy. (Piao et al., 2017) Thus, the Fenton reaction can enhance the anticancer efficacy by supplying O_2_ to the hypoxic TME, when used with other anticancer methods. Additionally, iron ions exert a crucial role in the process of ferroptosis, where lipid ROS level increases due to the suppression of system xc- and glutathione (GSH) synthesis. (Liu et al., 2018) GSH is the major endogen antioxidant system that leads to a decrease of ROS. (Cajas et al., 2020) Herein, Fenton reaction-based nanomaterials are of great importance in lung cancer therapy. DCFH-DA fluorescence intensity assays showed that PPy@Fe_3_O_4_ NPs significantly increased ROS levels generated from A549 cells under the conditions of H_2_O_2_. We also found that the PPy@Fe_3_O_4_ NPs promoted apoptosis of the cells of lung cancer induced by H_2_O_2_, as revealed by the Annexin V-FITC/PI assay and Calcine-AM/PI test. Therefore, the results of these experiments draw the following conclusion that PPy@Fe_3_O_4_ NPs could enhance antitumor effects through ROS damage.

Migration and invasion are two essential features of tumor metastasis. In metastasis, cancer cells invade and migrate from the primary site to the extracellular matrix and surrounding basement membrane, which further colonize the distant site. (Albuquerque et al., 2021) Therefore, the inhibition of migration and invasion could suppress the progression of lung cancer and extend the survival of patients. In our research, transwell chambers were established to confirm the inhibitory efficacy of PPy@Fe_3_O_4_ NPs on A549 cell migration. The results showed that all PPy@Fe_3_O_4_ NPs significantly suppressed A549 cell migration. Moreover, tumor metastasis is a very complicated process that involves the interaction of various genes and proteins, especially MMPs. MMPs have been reported as proteolytic enzymes with the capacity of degrading the extracellular matrix components and other secreted proteins of the lungs. (Gonzalez-Avila et al., 2019) And they are also associated with endothelial basement membrane destruction. The crack in the basement membrane allows cells to enter the circulation, which transports the tumor cells to the distant extravasation site. (Bonomi, 2002) MMP2, MMP9 and MMP13 are prominent enzymes that promote tumor cell invasion and migration and regulate the progress of epithelial-to-mesenchymal transition (EMT), which is a transformation process of the epithelial cells to the mesenchymal cells and can promote cancer metastasis. (Scheau et al., 2019) Meanwhile, the levels of these proteins all had been reported to be elevated in certain lung cancer patients. (Li et al., 2019; Han et al., 2020) Here, our results indicated that MMP2, MMP9 and MMP13 were manifestly downregulated by PPy@Fe_3_O_4_ NPs. Thus, these findings suggested that PPy@Fe_3_O_4_ NPs suppressed human lung cancer cell metastasis.

## Conclusion

In our study, we synthesized PPy@Fe_3_O_4_ NPs and demonstrated the safety and effectiveness of the PPy@Fe_3_O_4_ NPs *in vivo* and *in vitro*. The PPy@Fe_3_O_4_ NPs exhibited excellent synergistic effects of PTT and CDT, which could inhibit the growth and metastasis of lung cancer cells efficiently. Additionally, the PPy@Fe_3_O_4_ NPs was remarkable MRI contrast agents for tumors. However, the underlying mechanisms of tumor growth and metastasis need to be further discussed. Taken together, our study revealed the potential of PPy@Fe_3_O_4_ NPs to develop a new therapeutic strategy for NSCLC.

## Data Availability

The original contributions presented in the study are included in the article/Supplementary Material, further inquiries can be directed to the corresponding authors.

## Abbreviation

NSCLC, non-small cell lung cancer; NPs, nanoparticles; PPy, polypyrrole; MRI, magnetic resonance imaging; PTT, photothermal therapy; CDT, chemodynamic therapy; H_2_O_2_, hydrogen peroxide; ECM, extracellular matrix; TME, tumor microenvironment; MMPs, matrix metalloproteinases; PVA, Polyvinyl alcohol; TEM, transmission electron microscopy; FTIR, Fourier-Transformed Infrared; XRD, X-ray powder diffraction; NIR, near-infrared; CCK-8, cell counting kit-8; DCFH-DA, dichlorofluorescein-diacetate; ROS, reactive oxygen species; PBS, phosphate buffered saline; CLSM, Confocal laser scanning Microscopy; PI, propidium iodide; PVDF, polyvinylidene difluoride; HRP, horseradish peroxidase; TBST, Tris-buffered saline-Tween20; ECL, enhanced chemiluminescence; GSH, glutathione; EMT, epithelial-to-mesenchymal transition.
