# Supplementary material for: PPy@Fe3O4 Nanoparticles Inhibit Tumor Growth and Metastasis Through Chemodynamic and Photothermal Therapy in Non-small Cell Lung Cancer
Source: Front Chem. 2021 Nov 8;9:789934. doi: 10.3389/fchem.2021.789934 (PMC8606671; doi:10.3389/fchem.2021.789934)
Supplement: Supplementary file 1 [file DataSheet1.docx]

Supplementary Material

# Methods:

## Characterization of PPy@Fe_3_O_4_ NPs

The FTIR spectra were recorded on a FTIR spectrometer (Spectrum 2000; Perkin-Elmer, Waltham, MA, USA).

## Cell culture and animals

All animal experiments was approved by the Experimental Animal Center of Shanghai Jiaotong University. All nude mice were group bred under a 12-hour light/12-hour dark cycle with liberal supply of food and drink. The room temperature was set between 18℃ to 25℃, and the humidity was maintained to 60%. The nude mice were allowed to acclimate for one week before the start of experiments. Human lung adenocarcinoma A549 cells in this experiment were provided from the Cell Bank of the Chinese Academy of Sciences. A549 cells were incubated in RPMI 1640 medium containing 10% fetal bovine serum (FBS) and 1% Penicillin-Streptomycin. Meantime, all cultures were carried out at 37℃ in a humidified atmosphere of 5% carbon dioxide (CO_2_) and 95% air. Cells were passaged by trypsinization when the confluence reached 80%, and we changed the medium every two days.

## Cytotoxicity assay

The relative cell viability was calculated according to the following equation: Cell viability(%)=(OD experimental/OD control)*100%.

The photothermal conversion efficiency was calculated by the following equation：$ŋ$=$\frac{hS(T_{max}-T_{surr})-Q_{Dis}}{I(1-{10}^{{-A}_{808}})}$

where h is the heat transfer coefficient, s is the surface area of the container, T_max_ is the maximum steady-state temperature of the sample solution, T_surr_ is the ambient surrounding temperature, Q_Dis_ is the heat input due to light absorption by the solvent and container, I is the laser power, and A_808_ is the absorbance of the sample solution at 808 nm.

# Supplementary Figures


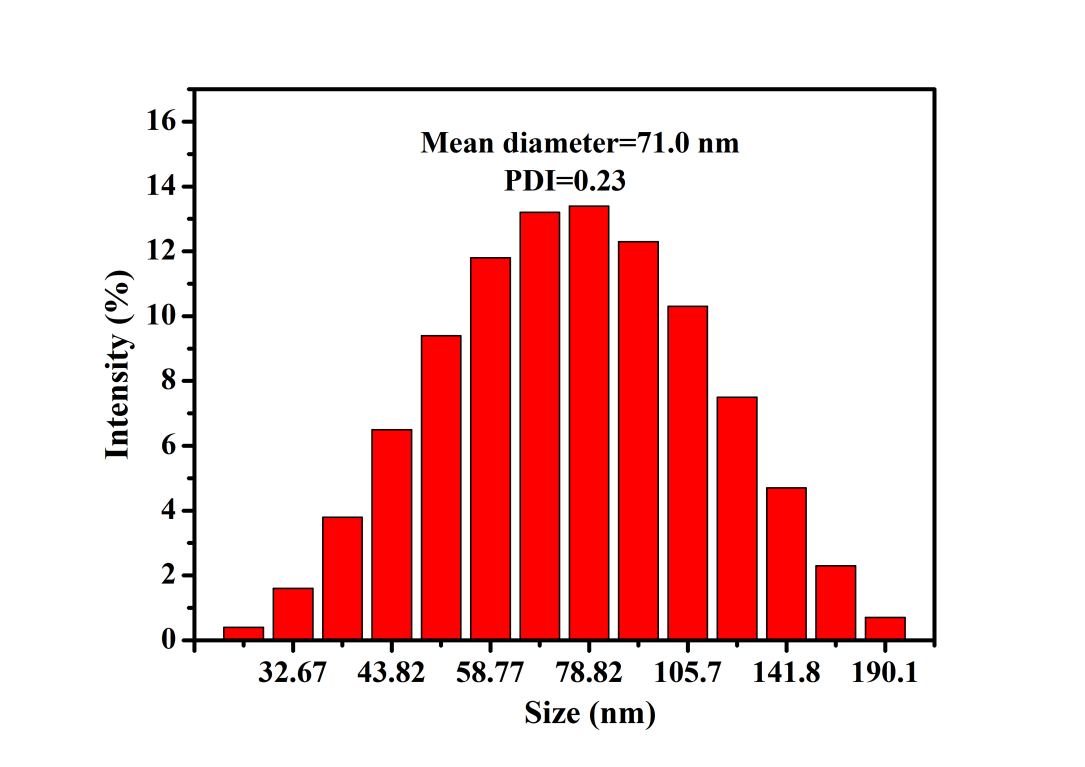


**Supplementary Figure 1.** The mean diameter of PPy@Fe_3_O_4_ nanoparticles is 71.0 nm.


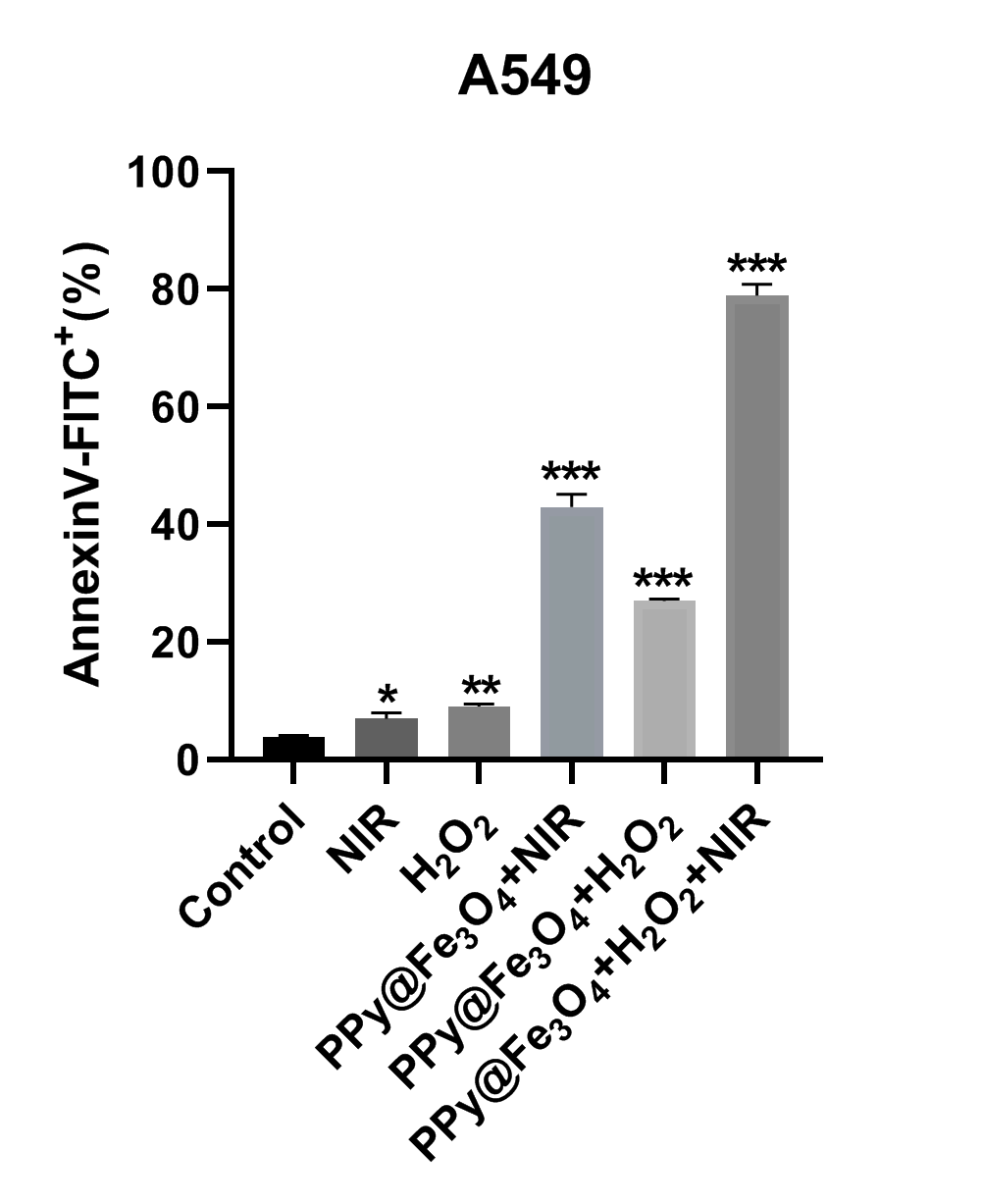


**Supplementary Figure 2.** The proportion of apoptosis in different groups.
